# Experimental and Theoretical Studies of Methyl Orange Uptake by Mn–Rich Synthetic Mica: Insights into Manganese Role in Adsorption and Selectivity

**DOI:** 10.3390/nano10081464

**Published:** 2020-07-26

**Authors:** Mohamed A. Barakat, Ali Q. Selim, Mohamed Mobarak, Rajeev Kumar, Ioannis Anastopoulos, Dimitrios Giannakoudakis, Adrián Bonilla-Petriciolet, Essam A. Mohamed, Moaaz K. Seliem, Sridhar Komarneni

**Affiliations:** 1 Department of Environmental Sciences, Faculty of Meteorology, Environment and Arid Land Agriculture, King Abdulaziz University, Jeddah 21589, Saudi Arabia; mabarakat@gmail.com (M.A.B.); olifiaraju@gmail.com (R.K.); 2 Central Metallurgical R & D Institute, Cairo 11421, Egypt; 3 Faculty of Earth Science, Beni-Suef University, Beni Suef 62511, Egypt; aliselimq@gmail.com; 4 Physics Department, Faculty of Science, Beni-Suef University, Beni Suef 62511, Egypt; saidan2011@yahoo.com; 5 Department of Chemistry, University of Cyprus, P.O. Box 20537, Nicosia Cy-1678, Cyprus; anastopoulos_ioannis@windowslive.com; 6 Institute of Physical Chemistry, Polish Academy of Sciences, Kasprzaka 44/52, 01-224 Warsaw, Poland; dagchem@gmail.com; 7 Departamento de Ingeniería Química, Instituto Tecnológico de Aguascalientes, Aguascalientes 20256, Mexico; petriciolet@hotmail.com; 8 Department of Ecosystem Science and Management and Materials Research Institute, 204 Energy and the Environment Laboratory, The Pennsylvania State University, University Park, PA 16802, USA; Komarneni@psu.edu

**Keywords:** hydrothermal synthesis, Mn–mica, methyl orange, statistical physics adsorption modeling

## Abstract

Manganese–containing mica (Mn–mica) was synthesized at 200 °C/96 h using Mn–carbonate, Al–nitrate, silicic acid, and high KOH concentration under hydrothermal conditions. Mn–mica was characterized and tested as a new adsorbent for the removal of methyl orange (MO) dye from aqueous solutions. Compared to naturally occurring mica, the Mn–mica with manganese in the octahedral sheet resulted in enhanced MO uptake by four times at pH 3.0 and 25 °C. The pseudo–second order equation for kinetics and Freundlich equation for adsorption isotherm fitted well to the experimental data at all adsorption temperatures (i.e., 25, 40 and 55 °C). The decrease of Langmuir uptake capacity from 107.3 to 92.76 mg·g^−1^ within the temperature range of 25–55 °C suggested that MO adsorption is an exothermic process. The role of manganese in MO selectivity and the adsorption mechanism was analyzed via the physicochemical parameters of a multilayer adsorption model. The aggregated number of MO ions per Mn–mica active site (n) was superior to unity at all temperatures signifying a vertical geometry and a mechanism of multi–interactions. The active sites number (*D*_M_) of Mn–mica and the total removed MO layers (*N*_t_) slightly changed with temperature. The decrease in the MO adsorption capacities (*Q_sat_* = *n·D_M_*·*N*_t_) from 190.44 to 140.33 mg·g^−1^ in the temperature range of 25–55 °C was mainly controlled by the *n* parameter. The results of adsorption energies revealed that MO uptake was an exothermic (i.e., negative Δ*E* values) and a physisorption process (Δ*E* < 40 kJ mol ^−1^). Accordingly, the adsorption of MO onto Mn–mica was governed by the number of active sites and the adsorption energy. This study offers insights into the manganese control of the interactions between MO ions and Mn–mica active sites.

## 1. Introduction

Mica is a layered silicate and belongs to phyllosilicate group of minerals and it has a 2:1 layered structure of two silica tetrahedral sheets [2T] sandwiching one alumina octahedral sheet [O], i.e., T: O:T structure. The mica minerals are more common in igneous rocks, especially the intrusive type, and they have different chemical compositions and properties. In addition to the natural micas, synthetic micas of different compositions can be prepared by different techniques such as topotactic [1], hydrothermal [2] and solid state [3] methods. High pressure, temperatures, and long interaction times are the main drawbacks of these preparation methods [2]. Muscovite [KAl_3_Si_3_O_10_ (OH)_2_] is a dioctahedral Al–rich clay mineral and one of the dominant minerals of the mica family and phlogopite [KAlSi_3_Mg_3_O_10_(OH)_2_] is a trioctahedral magnesium rich end member. Shirozulite, a Mn-dominant trioctahedral mica, is formed by the interaction between rhodochrosite (MnCO_3_) mineral in tephroite–rhodochrosite ore in contact with a Ba-bearing, K-feldspar [4]. A naturally occurring muscovite mica from Egypt with little or no Mn^2+^ was used to prepare pure cancrinite phase [5] In addition, some studies showed that muscovite has a capacity to remove several types of dissolved metals such as arsenic, copper, cadmium, and lead [6]. Furthermore, a composite of muscovite and phillipsite was prepared and utilized for phosphate and ammonium adsorption from contaminated aqueous solutions [7]. These studies have highlighted the potential applications of mica-based adsorbents in water treatment and purification. 

Textile, plastic, paper and cosmetic industries are associated with the discharge of organic dye contaminants such as methyl orange (MO) into the aquatic environment. Low concentrations of this azo dye in water leads to the deterioration of sunlight and oxygen dispersion [8,9]. In addition, MO is considered as a cancer–causing compound due to the existence of aromatic rings in its structure [10]. Therefore, precipitation, flocculation/coagulation, membrane filtration, photocatalysis, and adsorption were applied to treat the MO–polluted water [11]. Water remediation through the adsorption process was touted to be simple, promising, and economic [12]. Numerous adsorbents such as calcined coffee waste, carbon nanotubes, chitosan/Al_2_O_3_/Fe_3_O_4_, activated carbon/iron oxide, activated clay, and MCM–48 silica/ rice husk were utilized for MO uptake [12]. These types of adsorbents have active adsorption sites that can facilitate the removal of organic contaminants, including the tested MO dye.

Generally, the application of modeling tools in description and interpretation of the experimental data is very important because modeling will consider the physicochemical parameters involved in the adsorbate–adsorbent interface [13]. Freundlich and Langmuir (the dominant traditional adsorption models) have no deep theoretical bases to provide a complete interpretation for the adsorption mechanism [14,15]. The Langmuir assumption that implies the formation of one layer of the removed adsorbates on the tested adsorbent surface has been reported as the main drawback of the classical adsorption models [16,17]. By contrast, the adsorption modeling via the statistical physics concepts can provide reliable results to explain macroscopically and microscopically the adsorption mechanism [15,16].

The interaction between manganese oxides and raw materials was recommended as a favorable strategy in maximizing the value of these natural sources for water remediation [18,19]. Manganese–rich minerals (i.e., manganese is present in the octahedral sheet of synthetic clays such as smectite [20] and mica [2,21]. Without the addition of manganese carbonate to a mixture of KOH and aluminosilicate, synthetic mica was not produced (i.e., MnCO_3_ is essential to obtain mica) by hydrothermal treatment [2]. Manganese carbonate can be selected as a Mn source to prepare these aluminosilicates (smectite and mica) because this precursor can control the manganese oxidation state [2,20]. Moreover, it has been reported that potassium ions have lower hydration energy than that of sodium ions and, thus, K^+^ ions are suitable for the preparation of micas [22]. Using metakaolin as an aluminosilicate source resulted in the formation of sharp Mn–mica peaks at 200 °C/48 h regardless of the concentration of KOH [2]. By contrast, the aluminosilicate chemical sources (silicic acid and Al nitrate) gave poorly–crystalline mica peaks at 200 °C/48 h using a high KOH concentration [2]. 

The effect of KOH concentration and increasing the treatment time to 96 h has not been previously reported in the formation of well-crystallized Mn-rich mica phase. In addition, the adsorption capacity of Mn–mica for dyes has not been reported so far. Therefore, the objectives of this study were to (a) identify the critical concentration of K_2_O to prepare a well–crystallized Mn–mica with a high content of manganese, (b) characterize and test the uptake performance of the prepared Mn–mica for MO dye at varied conditions, (c) achieve new insights into the role of manganese in enhancing the adsorption capacity of MO dye, (d) use different models in describing the kinetics and isotherms of adsorption from the obtained experimental results, and (e) propose a deeper theoretical understanding for the adsorption process by determining the steric and energetic parameters of the advanced multilayer adsorption model.

## 2. Materials and Methods

### 2.1. Materials

Synthetic Mn–Rich mica was prepared using different chemicals such as KOH pellets (EM Science, purity >85%) as alkali hydroxide, silicic acid (Sigma-Aldrich, St. Louis, MO, USA, purity > 98%), and distilled water. Silicic acid (SiH_4_O_4_), Al–nitrate (Al (NO3)_3_. 9H_2_O, and manganese carbonate (MnCO_3_) were utilized as Si, Al, and Mn sources, respectively. Methyl orange (MO, C_14_H_14_N_3_NaO_3_S, Fluka, Buchs, Switzerland) was selected as an organic adsorbate in this study. To achieve the needed pH, of starting precursors, pH was adjusted by adding 0.01 M of HCl and NaOH solutions followed by measuring pH using a pH meter (Hanna, HI 9025). A stock solution (1000 mg·L^−1^) of MO was first prepared and the needed concentrations for the adsorption experiments were prepared by the addition of distilled water to the stock solution.

### 2.2. Synthesis of Mn–Rich Mica

A series of experiments were carried out to find the best concentration of K_2_O necessary to obtain Mn–Rich mica with high crystallinity. Table 1 summarizes the gel compositions with different concentrations of KOH, which were used to synthesize Mn–mica. Each mixture (gel) was transferred to a Teflon cup of a stainless steel hydrothermal vessel (125 mL capacity) and treated in an oven at 200 °C for different times of up to 96 h. After hydrothermal treatment, the solid and liquid phases were collected in clean centrifuge tubes (50 mL) and the solids were separated from solutions by centrifuging. Then, distilled water and ethanol were used five times to remove the excess of KOH in solution. The washed solid was dried at 70 °C/24 h and kept in a container for characterization and further MO adsorption experiments.

### 2.3. Characterization of Mn–mica

Different characterization techniques were used to investigate the composition and morphology of the synthesized Mn–mica. A Philips APD–3720 diffractometer with Cu Kα radiation (wavelength (λ) = 1.54 A) was used to determine the X–ray diffraction (XRD) pattern of Mn–mica in the two theta range of (2–40°) with a scan rate of 2°/min. The morphological features of Mn–mica were studied through scanning electron microscope (SEM, JSM-6700F, JEOL, Tokyo, Japan) and transmission electron microscope, TEM, FEI Techni F20, Tokyo, Japan). Fourier transform infrared (FT-IR) analysis was employed to detect the functional groups of Mn–mica in the range of 400–4000/cm (Bruker FT/IR-2000 Spectrometer, Berlin, Germany). The value of zero charge point (pH_ZCP_) of Mn–mica was identified using previously established procedure [23] as follow: A solution of 0.1 M NaCl (5.85 g) was prepared and then 50 mL solutions of 0.1 M NaCl were added to clean beakers. The initial pH (pH_i_) of these solutions was adjusted from 2 to 10 by adding either 0.1 M NaOH or 0.1 M HCl solutions. The Mn–mica dose (0.1 g) was mixed with each 50 mL 0.1 M NaCl solution at 200 rpm for 48 h. The difference between the initial pH (pH_i_) and final pH (pH_f_), i.e., pH_f_–pH_i_ was plotted versus pH_i_. The point at which pH_f_–pH_i_ equal to zero was reported to be the value of zero charge point (pH_ZCP_). 

### 2.4. Effect of pH on Methyl Orange (MO) Uptake by Mn–mica

In order to evaluate the role of pH on MO uptake, adsorption experiments were performed in the pH range of 2.0–10.0 at temperature of 25 °C. However, the other parameters were kept constant (i.e., 50 mg of Mn–mica mass, 50 mg·L^−1^ of MO concentration, and 2 h of interaction time). After separation of liquid phases in all adsorption experiments by centrifuging, a double beam ultraviolet (UV)–visible absorption spectrometer (Shimadzu, UV 1601) was applied to determine MO (λ_max_ = 463 nm) concentrations. The MO uptake by the as-synthesized Mn–mica was compared with a natural mica containing only a little Mn under the same pH conditions [5]. The natural mica sample used for testing of MO uptake was collected from Eastern Desert in Egypt. The natural mica has a composition of SiO_2_ (48.19%), Al_2_O_3_ (31.75%), Fe_2_O_3_ (5.12%), MgO (0.71%), MnO (0.05%), K_2_O (8.49%), Na2O (0.66%), TiO_2_ (0.11%), and CaO (0.03%), and LOI (4.49%) as has been reported by [5]. 

### 2.5. MO Adsorption Kinetics Using Mn–mica 

MO adsorption kinetics were measured at three temperatures (25, 40, and 55 °C) using a mixture of 0.05 g of Mn–mica and 50 mL of MO with 50 mg·L^−1^ of dye concentration. The adsorbate solution (MO) and adsorbent (Mn–mica) were mixed at 200 rpm at varied time periods ranging from 5 to 480 min. The remaining MO concentration in solutions was measured after each time of adsorbate–adsorbent contact. The adsorption capacity ($q_{t}$) and the removal percentage (R%) of MO were determined at each temperature using Equations (1) and (2).

(1) $$q_{t}\left(\mathrm{mg}\;g^{-1}\right)=\left(C_{0}–C_{t}\right)\frac{V}{m}\;\left(1\right)$$

(2) $$R\;(\%)=\frac{100}{C_{0}}\left(C_{0}–C_{t}\right)\;\left(2\right)$$

where $C_{0}$ and $C_{t}$ (mg·L^−1^) are the initial and remaining concentrations of the studied dye after time (*t*), V is the dye solution volume (L) and m is the Mn–mica dose (g). The pseudo–first–order [24], the pseudo-second-order [25] and intra-particle diffusion [26]. Equations were applied to study the kinetics and the diffusion mechanism associated with the MO uptake onto Mn–mica adsorbent, see Table 2. 

### 2.6. Equilibrium Studies of MO Adsorption on Mn–mica

MO adsorption isotherms were quantified to characterize the Mn–mica performance and to apply traditional and statistical physics adsorption for analyzing the dye adsorption mechanism. Adsorption of MO on Mn–mica was carried out at pH 3.0 and different temperatures (25 °C, 40 °C and 55 °C). These experiments were conducted by mixing 50 mL of MO solutions (starting concentrations 20–120 mg·L^−1^) with 0.05 g of Mn–mica. The solid and liquid phases were mixed at 200 rpm for 120 min of interaction time using a SHO–2D (Germany) shaker. The adsorbed MO quantities at adsorption equilibrium (*q_e_*) were calculated as follows:

(3) $$q_{e}\left(\mathrm{mg}\;g^{-1}\right)=\left(C_{0}–C_{e}\right)\frac{\;V}{\;m}\;$$

where $C_{e}$ (mg·L^−1^) is the equilibrium concentration of dye MO.

All kinetics and equilibrium adsorption experiments were repeated three times and the results were averaged, where the experimental errors were of less than 4%.

### 2.7. Conventional and Advanced Dye Adsorption Modeling 

Table 3 displays the non–linear equations of Langmuir [27], Freundlich [28] and Dubinin–Radushkevich (D–R) [29] used as common classical models for fitting MO adsorption data on Mn–mica. The best fit of the conventional isotherm model was identified from the determination coefficient (*R*^2^) and Chi–square ($\chi^{2}$) values. To theoretically analyze the adsorption of MO on adsorbent Mn–mica, an advanced multilayer adsorption model (in its general form) was used as previously given [15]

(4) $$Q=n\;D_{M}\frac{F_{1}\left(c\right)+F_{2}\left(c\right)+F_{3}\left(c\right)+F_{4}\left(c\right)}{G\left(c\right)}\;$$

where

(5) $$F_{1}\left(c\right)=-\frac{2{\left(\frac{c}{c_{1}}\right)}^{2n}}{1-{\left(\frac{c}{c_{1}}\right)}^{n}}+\frac{{\left(\frac{c}{c_{1}}\right)}^{n}\left(1-{\left(\frac{c}{c_{1}}\right)}^{2n}\right)}{{\left(1-{\left(\frac{c}{c_{1}}\right)}^{n}\right)}^{2}},$$

(6) $$F_{2}\left(c\right)=\frac{2{\left(\frac{c}{c_{1}}\right)}^{n}{\left(\frac{c}{c_{2}}\right)}^{n}\left(1-{\left(\frac{c}{c_{2}}\right)}^{n\;N_{2}}\right)}{1-{\left(\frac{c}{c_{2}}\right)}^{n}},$$

(7) $$F_{3}\left(c\right)=-N_{2}\frac{{\left(\frac{c}{c_{1}}\right)}^{n}{\left(\frac{c}{c_{2}}\right)}^{n}{\left(\frac{c}{c_{2}}\right)}^{n\;N_{2}}}{1-{\left(\frac{c}{c_{2}}\right)}^{n}},$$

(8) $$F_{4}\left(c\right)=\frac{{\left(\frac{c}{c_{1}}\right)}^{n}{\left(\frac{c}{c_{2}}\right)}^{2n}\left(1-{\left(\frac{c}{c_{2}}\right)}^{n\;N_{2}}\right)}{{\left(1-{\left(\frac{c}{c_{2}}\right)}^{n}\right)}^{2}},$$

(9) $$G\left(c\right)=\frac{\left(1-{\left(\frac{c}{c_{1}}\right)}^{2n}\right)}{1-{\left(\frac{c}{c_{1}}\right)}^{n}}+\frac{{\left(\frac{c}{c_{1}}\right)}^{n}{\left(\frac{c}{c_{2}}\right)}^{n}\left(1-{\left(\frac{c}{c_{2}}\right)}^{n\;N_{2}}\right)}{{\left(1-{\left(\frac{c}{c_{2}}\right)}^{n}\right)}^{2}},$$

where the parameter *n* indicates the number of attached MO molecules per active site of the adsorbent, *D_M_* is the active sites density of Mn–mica, *C*_1_ and *C*_2_ are the concentrations at half–saturation (i.e., *C*_1_ is associated with the first layer, while *C*_2_ is restricted to *N*_2_ layers of the removed dye molecules). 

This model supposed that the adsorption of MO ions was governed by dissimilar uptake energies, which frequently resulted in the development of a controlled number of the studied azo dye layers [16,30]. The primary adsorption energy is related to the interaction between the first adsorbed MO layer (fixed number) and the Mn–mica surface (i.e., dye MO–Mn-mica interaction). On the other side, the second removal energy describes the dye–dye interaction in the adsorbed MO layers (i.e., MO–MO interaction). Consequently, the full number of the removed MO layers is given by 1 + *N*_2_. The calculated *N*_2_ value can be equal to zero, unity or higher than 1, which indicated a monolayer, double–layer and multilayer adsorption process, respectively [23]. Numerous scenarios for this model were suggested to study the MO uptake on Mn–mica as follows [16].

❖ Status 1: *n* and *N*_2_ are free adjustable parameters (i.e., multilayer).
❖ Status 2: *n* is a free adjustable parameter and *N*_2_ = zero (fixed) (i.e., a monolayer).
❖ Status 3: *n* is a free adjustable parameter and *N*_2_ =1 (fixed) (i.e., double–layer)
❖ Status 4: *n* is a free adjustable parameter and *N*_2_ =2 (fixed) (i.e., triple–layer)
❖ Status: *n* = unity (fixed) and *N*_2_ = zero (fixed) (i.e., Langmuir model)

## 3. Results and Discussions

### 3.1. Characterization of Mn–mica 

**Figure 1:**
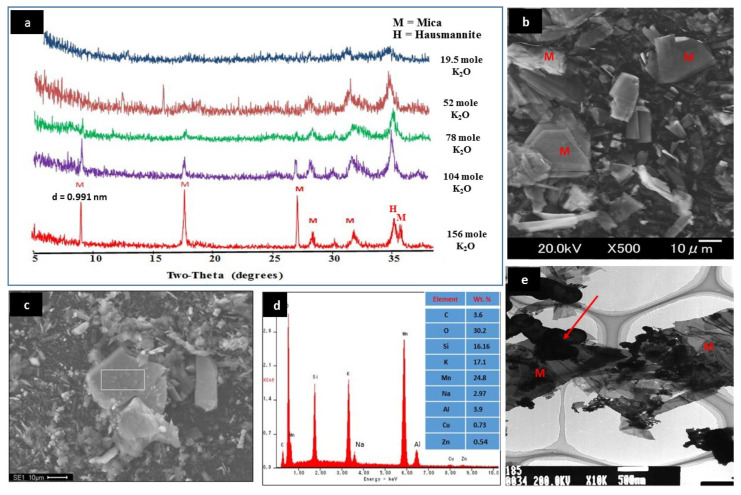
Effect of K_2_O moles on the crystallization of Mn–mica as revealed by XRD patterns (**a**), scanning electron microscopy (SEM) (**b**), energy-dispersive X-ray spectroscopy (EDX) (**c**,**d**), and transmission electron microscopy (TEM) (**e**) results of the well-crystallized Mn–mica using 156 moles of K_2_O.

Figure 1a displays the XRD pattern of each gel composition listed in Table 1. The XRD results were mainly attributed to the change of concentrations of KOH, because all the other conditions were kept constant (see Table 1). By using the highest concentration of KOH (i.e., 156 K_2_O moles), the detected sharp peaks at 2θ = 8.93°, 17.84°, 26.86°, and 36.05° indicated the (002), (004), (006), (008) lattice planes of mica phase [7]. On the other hand, the use of 104 and 78 moles of K_2_O resulted in a significant decrease in the intensities of these planes. In the gel composition containing either 52 or 19.5 K_2_O moles, the main peaks of Mn–mica disappeared. Therefore, the high KOH concentration was found to be an important factor in the synthesis of well-crystallized Mn–mica (Figure 1a). In addition, the c-dimension, i.e., (001) spacing of Mn–mica was determined to be 0.991 nm (see the first peak of the synthetic mica in Figure 1a). Mn is mostly divalent as determined by the 060 spacing representing trioctahedral nature with divalent Mn ions in one of our previous study (see reference [21]). In the prepared Mn–mica, divalent Mn replaces Mg positions in a phlogopite mica (KSi_3_AlMg_3_O_10_OH_2_) composition. Furthermore, small amount of hausmannite (Mn_3_O_4_) was observed as a co–crystallized phase along with mica in all products of different KOH concentrations. More detailed studies are necessary, however, to understand the roles of Mn and Al, in the crystallization of Mn–mica. 

Based on the XRD results, Mn–mica prepared using the gel composition of 156 K_2_O, 13.05 MnO, 16.01 SiO_2_, 1.00 Al_2_O_3_, 1388 H_2_O was selected for more investigations by SEM, Fourier transform infrared (FTIR) spectroscopy, energy-dispersive X-ray spectroscopy (EDX), and TEM techniques. In addition, this well–crystallized mica was applied to carry out all MO adsorption experiments. SEM result displays the presence of particles with flaky habit and clear sharp edges, confirming the synthesis of Mn–mica sheets (Figure 1b). The external surface area of this mica, which depend on particle size, is expected to be very small because of its micrometer particle size (see Figure 1b). The EDX analysis of the as-synthesized Mn–mica (Figure 1c,d) revealed the presence of oxygen (30.2%), silicon (16.16%), manganese (24.8%), aluminum (3.9%), and potassium (17.1%) as the main elements in the synthetic Mn–mica (Figure 1d). Ideal Mn-mica composition is as follows: KSi_3_AlMn_3_O_10_OH_2_. However, we determined the very approximate elemental composition by EDX (see Figure 1d).

**Figure 2:**
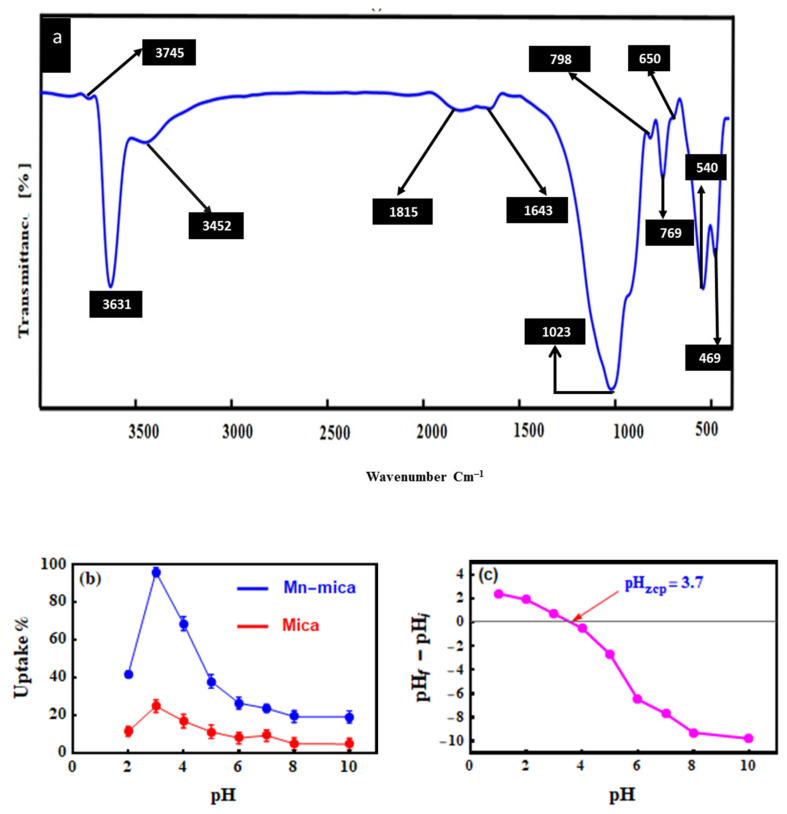
Infrared spectrum of the well crystallized mica showing the main absorption bands (**a**), effect of pH on the uptake of MO by the well-crystallized Mn–mica (**b**) and zero charge point of Mn–mica (**c**).

The FTIR spectrum of the synthetic Mn–mica shows the existence of several detectable bands (Figure 2a). The observed bands at about 3745 (very small band) and 3631 (very large band) cm**^−1^** could be associated with the stretching vibration of hydroxyl (–OH) groups in the octahedral sheet of Mn–mica [23,31]. The band detected at 3452 cm^−1^ was assigned to the O–H stretching of the silica group in the Mn–mica surface [32]. The formed bands at 1815 and 1643 cm^−1^ were corresponding to the carbonate CO_3_^2−^group vibration and the –OH deformation of adsorbed H_2_O molecules on the surfaces of the crystallized Mn–mica, respectively [5,31,33]. The identified band at about 1020 cm^−1^ is associated with the Si–O stretching vibrations [11]. The absorption bands at 650, 798, and 819 cm^−1^ could be related to the Mn–O lattice vibrations [34]. The bands at 540 and 469 cm^−1^ were related to the Si–O–Al and Si–O–Si bending vibrations, respectively [11]. 

### 3.2. Effect of pH on the MO Adsorption by Mn–mica

Figure 2b displays MO uptake using the well-crystallized Mn–mica adsorbent over the tested pH range (i.e., pH 2.0–10.0). Due to the high concentration of hydrogen ions (H^+^) in solution at pH 2.0, the active removal sites of the Mn–mica such as –OH_2_^+^, Si–OH^+^, and MnOH_2_^+^ were protonated. Therefore the electrostatic repulsion between the protonated Mn–mica functional groups and the protonated MO (i.e., the MO has net positive charge at pH = 2.0) caused a decrease of dye uptake [35]. By contrast, at pH 3.0, the maximum MO removal percentage (95.93%) could be associated with the strong electrostatic interaction between the anionic MO (i.e., MO ionization degree reach its maximum at pKa = 3.46) and the protonated sites of Mn–mica adsorbent. Furthermore, the molecular structure of the MO contains the S, N and O atoms which may form the hydrogen bonding with the –OH/–OH_2_^+^ groups of the Mn–mica surface [11]. In addition, the van der Waals force was considered as an additional influence resulting in improving the MO uptake [11]. By increasing the pH values from 4.0 to 10.0, the uptake of MO was significantly decreased from 68.49 to 18.96%, respectively (Figure 2b). The decrease of MO uptake at higher pH values could be related to the competition between the abundant hydroxyl ions and MO anions. The deprotonated groups of Mn–mica do not permit the adsorption of anionic MO dye due to the electrostatic repulsion. The H–bonding between the Mn–mica adsorbent and MO dye was not formed due to the deprotonation of Mn–mica active sites and, thus, the low percentages of MO uptake at higher pH were mainly related to van der Waals force. Moreover, the value of pH_ZCP_ of tested Mn–mica was 3.7 (Figure 2c) thus indicating that the adsorbent surface will capture MO dye molecules more efficiently at pH 3.0. 

Thus, the MO adsorption experiments in the current study were carried out in solution pH = 3.0. In addition, the uptake of MO was determined also with natural mica in the entire pH range (see Figure 2b) for comparison with Mn-mica. The adsorption capacity of Mn–mica for MO capture was much higher than that of the natural mica serving as a control as it has little or no Mn. Thus, the presence of manganese in the octahedral sheet of the synthetic mica may have played an important role in enhancing the removal efficiency for MO dye.

### 3.3. Contact Time Effect on MO Uptake by Mn–mica

**Figure 3:**
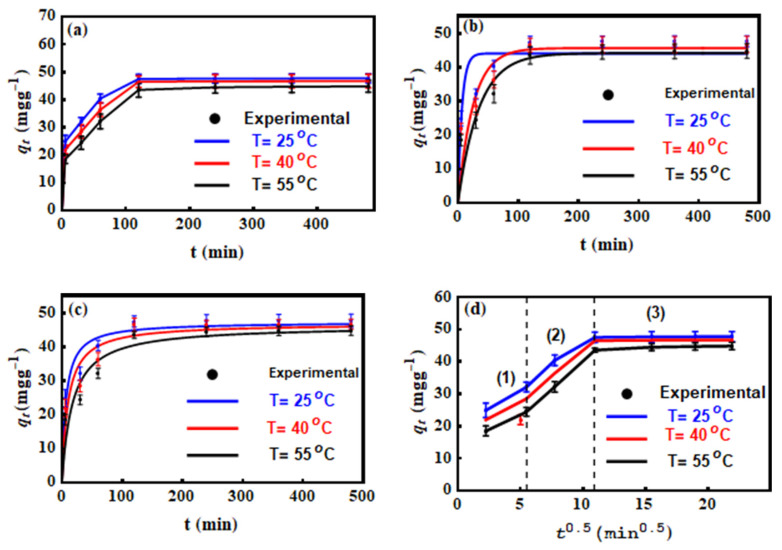
Kinetic studies of MO uptake by the Mn-mica, effect of contact time (**a**), pseudo-first order model (**b**), pseudo-second order model (**c**) and intra-particle diffusion model (**d**) at different temperatures.

The adsorption of MO on Mn–mica was studied at different contact times (5 to 480 min) and three solution temperatures (25 °C, 40 °C and 55 °C), see Figure 3a. The adsorbed MO amounts ($q_{t}$) were 24.89, 21.95, and 18.48 mg·g ^−1^ at 25, 35 and 45 °C, respectively, at early contact times (i.e., up to 5 min) of the removal process. The removed MO amount in this period (i.e., 0–5 min) were high due to the significant number of Mn–mica functional groups available for dye adsorption [36]. From 5 to 120 min of adsorbate–adsorbent contact time, the $q_{t}$ values improved to 47.5, 46.52, and 43.54 mg·g^−1^ at the tested adsorption temperatures of 25, 35 and 45 °C, respectively. This increase could be related to the occupation of more Mn–mica surface active sites by MO and, furthermore, the movement of dye ions into the intraparticle pores (i.e., intra–particle diffusion dye transfer) [37,38,39,40]. There is no measurable porosity because any mica including Mn–mica by definition is non-porous unless it is an aggregated particle with many small crystals [2]. From 120 to 480 min, the $q_{t}$ values of adsorbent Mn–mica were nearly constant (Figure 3a) thus suggesting the achievement of the equilibrium adsorption stage. 

### 3.4. MO Adsorption Kinetics 

The non–linear plots of $q_{t}$ against time (Figure 3b,c) were used to determine the parameters of the used kinetics models (pseudo-first and pseudo-second order equations) and the results are listed in Table 4. The *R*^2^ values of the applied kinetic models are very close to one indicated that the adsorption data of MO uptake using Mn–mica at all temperatures were in good agreement with the pseudo-first-order as well the pseudo-second-order equations. Therefore, we can consider that the pseudo-first order kinetic model has also an effect on the MO adsorption process. On the other hand, the pseudo-second-order equation described well (i.e., *R*^2^ values are little higher) as compared to the pseudo-first-order kinetic model. Additionally, the calculated and experimental *q_e_* values for the pseudo-second-order model were very similar, which confirm the applicability of this model for fitting the MO adsorption data [40]. Similar behavior was also reported in several published articles dealing with the uptake of water contaminates via the adsorption process [41,42].

**Figure 4:**
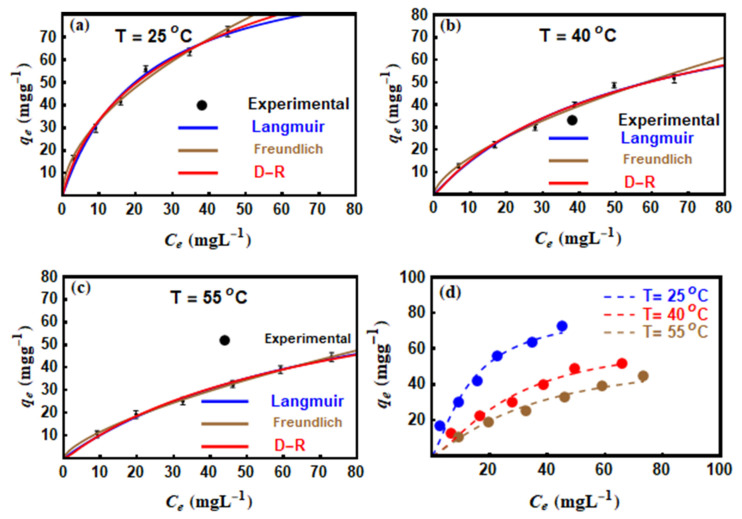
Isotherms of the adsorption of MO on adsorbent Mn-mica and their modeling with (**a**–**c**) traditional (i.e., Langmuir, Freundlich and D-R) and (**d**) statistical physics models.

The intra-particle diffusion model was applied to obtain an idea for the diffusion mechanism of MO in the adsorbent (Mn–mica) pores and also, to identify the rate–controlling step during the adsorption [12]. The plot of $q_{t}$ versus $t^{1/2}$ (Figure 3d) was utilized to find the parameters $k_{p}$ and *C* of this model as listed in Table 4. This model exhibited three stages in the whole time range of 5–480 min as presented in Figure 4d. These stages corresponded to the external mass transfer (stage 1), pore–diffusion (stage 2) and equilibrium (stage 3). Accordingly, the MO adsorption using Mn–mica was controlled by a mixture of film diffusion and intra-particle diffusion mechanisms [36]. This kinetic model indicated that MO ions were adsorbed on the surface as well as in the formed pores of the developed Mn–mica. 

### 3.5. Dye Adsorption Modelling with Traditional Equations 

At all temperatures, the non–linear methods of Langmuir, Freundlich, and D–R are shown in Figure 4 and their determined parameters are listed in Table 5. In general, all the three applied models were found to be appropriate to fit the MO adsorption results with $R^{2}$ values close to unity. At 25, 35 and 45 °C, the maximum uptake capacities ($q_{max}$) calculated through the Langmuir equation were 107.3, 97.36, and 92.76 mg·g^−1^ for MO, respectively. The decrease of $q_{max}$ with increasing temperature from 25 to 55 °C revealed the exothermic interaction between the tested azo dye and Mn–mica adsorption sites [33]. Correspondingly, the $K_{F}$ values of the Freundlich model also decreased with temperature (Table 5) confirming the exothermic nature of dye adsorption [43]. Furthermore, the variation of 1/n values from 0.532 to 0.679 within the temperature range of 25–55 °C (i.e., 1/n < 1) reflecting the positive MO removal at low concentrations [14]. The E values calculated via the D–R model ($E=\frac{1}{\sqrt{2\beta }}$) were less than 8.0 kJ mol^−1^ suggesting the physical adsorption of MO on the Mn–mica adsorbent [15]. Moreover, the values of $\chi^{2}$ showed that the Freundlich equation was the best for fitting MO adsorption data (i.e., $\chi^{2}$ presented the minimum values at all temperatures, see Table 5). This behavior indicated the presence of different adsorption sites on the Mn–mica surface where a MO uptake followed a complex behavior [11]. 

### 3.6. Dye Adsorption Modelling with Advanced Equations 

The suitability of the advanced multilayer model to fit the experimental MO adsorption data at 25, 40 and 55 °C is given in Figure 4d where *R*^2^ values were close to unity. Therefore, the steric parameters (*n*, *D_M_*, *N*_t_) and the removed amounts at saturation (*Q_sat_* = *n·D_M_*·*N*_t_) were calculated. Besides, the adsorption energetic (Δ*E*) related to the interaction between MO and Mn–mica were determined. The calculated physicochemical parameters (steric and energetic) controlling the adsorption mechanism are deeply discussed through the applied multilayer model.

#### 3.6.1. The *n* Parameter

**Figure 5:**
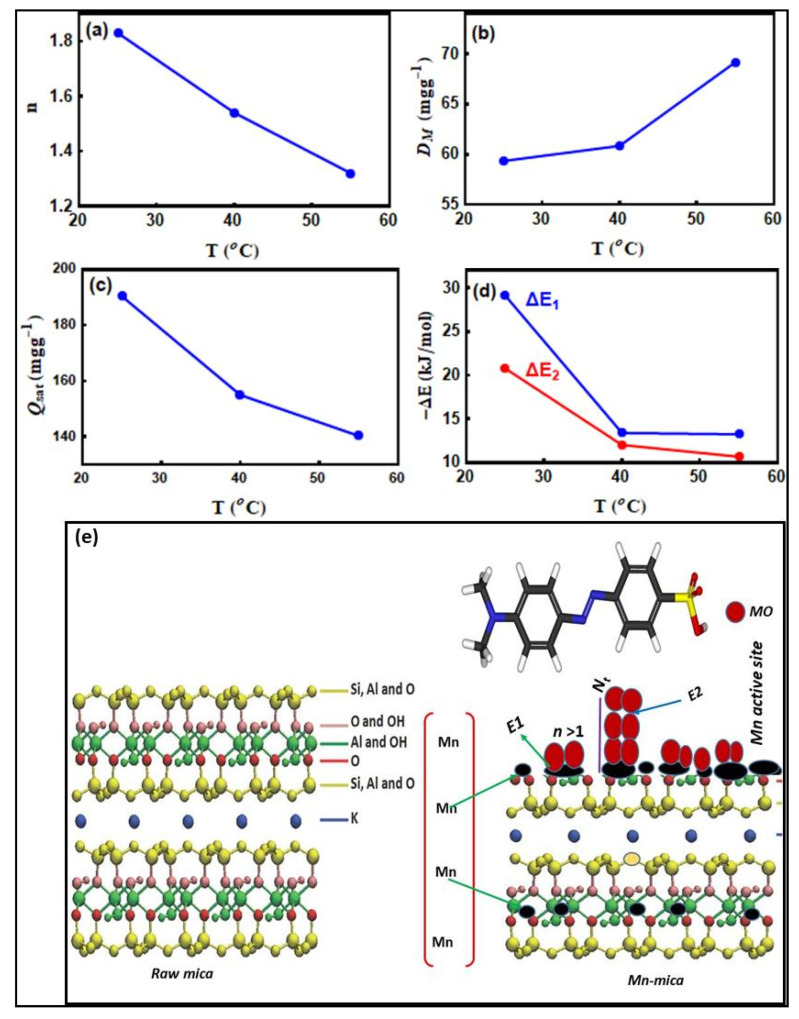
Evolution of *n* (**a**), *D_M_* (**b**), *Q*_sat_ (**c**), ΔE (**d**) parameters as a function of temperature and the possible adsorption mechanism for MO on Mn-mica (**e**).

The involved physicochemical parameter *n* in the multilayer model was used to modify the hypothesis of the Langmuir adsorption model. The value of this parameter can be = 1 (i.e., Langmuir assumption), greater or smaller than unity, which reflects different scenarios of the adsorbent behavior. This parameter plays a significant role in clarifying the geometry (vertical or horizontal) of the adsorbed MO ions on the Mn–mica surface. The vertical or horizontal position of the adsorbed MO ions are related to n with a value greater or lower than unity, respectively. Additionally, the adsorption mechanism of the dye MO on Mn–mica could be multi-docking in the case of *n* < 1 or multi–ionic in the scenario of *n* > 1 [13,14,15,16,17]. Therefore, numerous active sites of the Mn–mica can adsorb one MO ion if *n* < 1, while one adsorption site of this adsorbent can capture many dye ions when *n* > 1 [33]. Figure 5a displays the variation of this parameter as a function of temperature and the resulting *n* values are listed in Table 6. It can be seen that the parameter *n* was 1.83, 1.54, and 1.32 at 25, 40, and 55 °C (i.e., they were superior to 1). Therefore, the vertical geometry and multi–ionic mechanism were involved throughout the interaction between MO ions and Mn–mica active sites [15]. This result suggested that several MO ions interacted with one active site of the adsorbent Mn–mica (i.e., multi–interactions mechanism) and thus, the adsorbed dye ions were vertically oriented [13,14,15,16]. The aggregation of MO dye ions in aqueous solution (i.e., before the interactions between the MO ions and the Mn–mica adsorbent surface) was the main reason for the presence of this vertical geometry in the adsorption processes [44]. MO dye has a small molecular size (1.2 nm) and it can be aggregated in solution due to its free movement, which facilities the MO–MO binding [45,46]. Since the *n* values were within the range of 1.3 (monomer: *n* ~ 1) and 1.83 (dimer: *n* ~ 2) (Figure 5a), the aggregated MO ions apparently interacted with one active adsorption site of the Mn–mica surface thus implying a non–parallel adsorption position. These selective receptor sites are anticipated to be associated with the presence of manganese ions in the Mn–mica structure.

#### 3.6.2. The *D_M_* Parameter

Figure 5b shows the difference of the Mn–mica active sites number (the *D_M_* parameter) with respect to solution temperature. At 25, 40, and 55 °C, the values of *D_M_* were 59.34, 60.85 and 69.18 mg·g^−1^ for MO as given in Table 6. The increase of the *D_M_* values could be linked to the decrease of the *n* parameter (generally, the *D_M_* and *n* parameters show a reverse trend) through the adsorption process. Normally, the adsorbate aggregation phenomenon caused an increase of the *n* parameter and, consequently, a decrease in the number of the occupied Mn–mica active sites (i.e., a decrease of *D_M_*). Thus, the low accumulation of MO ions onto the Mn–mica surface with temperature resulted in the involvement of new active sites of this adsorbent in the adsorption process.

#### 3.6.3. Total Number of the Adsorbed MO Layers (*N*_t_ = 1 + *N*_2_) 

The determination of the total number of adsorbate layers to provide a better understanding of the adsorption mechanism of the MO ions on Mn–mica was necessary [23,44]. *N*_t_ values were determined as 1.75, 1.65 and 1.53 for MO at 25, 40 and 55 °C, respectively (Table 6). The reduction of the *N*_t_ value from 1.75 to 1.53 with the increase of adsorption temperature could be related to the impact of thermal agitation [44]. This effect resulted in a disordered movement (i.e., unsettled state) of the adsorbed MO ions and, therefore, a decrease of the formed adsorbate layers. It can be observed that the parameters *n* and *N*_t_ have the same trend (i.e., both decreased with temperature) in the MO adsorption. Furthermore, the slight variation of the *N*_t_ values at all tested temperatures indicated that the role of this parameter to control the adsorption process was not significant. Therefore, the impact of parameter *N*_2_ (the number of the removed MO layers) in the adsorption mechanism could be discarded. 

#### 3.6.4. Adsorption Capacity of MO at Saturation (*Q_sat_* = *n·D_M_*·*N*_t_)

Calculation of the *Q_sat_* values is necessary to distinguish the uptake efficacy of Mn–mica for the MO removal from the polluted solutions at all adsorption temperatures. Figure 5c displayed the behavior of this parameter with increasing temperature and the corresponding results were given in Table 6. The *Q_sat_* values were 190.44, 154.99 and 140.33 mg·g^−1^ for MO at 25, 40 and 55 °C, respectively. The decrease of the *Q_sat_* values with temperature confirmed the exothermic interaction between MO ions and Mn–mica. The *Q_sat_* values displayed the same trend identified for the parameters *n* and *N*_t_ (i.e., these parameters decreased with temperature), see Figure 5 and Table 6. It has been explained in the previous section that the effect of *N*_t_ on the MO adsorption can be ignored and, therefore, the adsorption mechanism of this dye were mainly governed by the parameter *n*. Additionally, if it was assumed that *N*_2_ = zero, the results of n. *D*_M_ showed close values to the monolayer adsorption model, which reflected the matching between the experimental and theoretical data. 

### 3.7. Dye Adsorption Energy (ΔE) 

Calculation of dye adsorption energies is an important to attain a deep interpretation for the interaction between MO ions and Mn–mica active sites [14]. The determination of these adsorption energies was performed by the next equations (Equations (6) and (7)).

(10) $$C_{1}=C_{s}e^{-\frac{\Delta E_{1}}{RT}}$$

(11) $$C_{2}=C_{s}e^{-\frac{\Delta E_{2}}{RT}}$$

where $c_{1}$ and $c_{2}$ denote the concentrations at half-saturation and $c_{s}$ signifies the solubility of MO dye.

Figure 5d and Table 6 show the values of MO adsorption energies (Δ*E*) with respect to the adsorption temperature. The negative values of Δ*E* confirmed the exothermic nature of MO adsorption on Mn–mica. Moreover, the Δ*E* values for dye MO were less than 40 kJ mol ^−1^ (see Table 6), which suggested a physical adsorption process [44]. As expected, the values of ΔE_1_ were higher than those of ΔE_2_ at 25, 40, and 55 °C because ΔE_1_ was associated with the interactions between Mn–mica active sites and adsorbate MO, while ΔE_2_ reflected the MO–MO interactions. Energetically, the ΔE trend was found to be similar to that of *Q_sat_* with increments of temperature. Consequently, the adsorption mechanism of MO on Mn–mica was governed by steric (*n*) and energetic (ΔE) parameters as shown in Figure 5e.

## 4. Conclusions

Mn–mica was hydrothermally synthesized and tested as a new adsorbent for dye MO at different operating conditions. Grafting manganese in the octahedral sheet of this adsorbent actually improved its MO adsorption by more than 400% at solution pH 3.0. At temperatures of 25, 40 and 55 °C, The pseudo-second-order and Freundlich models described the MO adsorption on Mn–mica at all tested adsorption temperatures. Physicochemical parameters of the multilayer adsorption model were calculated and discussed. MO ions were arranged in a vertical position and governed by multi-interaction mechanisms. Sterically, the *n* parameter played the most important role in managing the adsorption process. The adsorption energies indicated that MO adsorption on Mn–mica was exothermic and related to physical interaction forces. From this study, Mn–mica is suggested as a potential adsorbent for the uptake of MO dye from water. Moreover, the applied advanced multilayer model provided a deeper understanding for the adsorption mechanism of this azo dye. 

## Figures and Tables

**Table 1:** Gel composition and X-ray diffraction (XRD) results for the preparation of Mn–mica at different KOH concentrations.

Gel Composition (Moles)	Temperature (°C)	Time (h)	XRD Result
(1)156 K_2_O, 13.05 MnO, 16.01 SiO_2_, 1.00 Al_2_O_3_, 1388 H_2_O	200	96	Sharp Mn–mica peak
(2)104 K_2_O, 13.05 MnO, 16.01 SiO_2_, 1.00 Al_2_O_3_, 1388 H_2_O	200	96	Weak Mn–mica peak
(3)78 K_2_O, 13.05 MnO, 16.01 SiO_2_, 1.00 Al_2_O_3_, 1388 H_2_O	200	96	Weak Mn–mica peak
(4)52 K_2_O, 13.05 MnO, 16.01 SiO_2_, 1.00 Al_2_O_3_, 1388 H_2_O	200	96	No Mn–mica peak
(5)19.5 K_2_O, 13.05 MnO, 16.01 SiO_2_, 1.00 Al_2_O_3_, 1388 H_2_O	200	96	No Mn–mica peak

**Table 2:** Kinetic models used to fit the adsorption of methyl orange (MO) on adsorbent Mn–mica.

Kinetic Model	Equation	Parameters	Refs.
Pseudo-first-order	$q_{t}=q_{e}\left(1-e^{-k_{1}t}\right)$	$q_{t}$ (mg·g^−1^): removed amount of MO at time t^.^$\;q_{e\;}$(mg·g^−1^): equilibrium adsorption uptake $k_{1\;}$(min^−1^): rate constant of the first-order adsorption.	[24]
Pseudo-second-order	$q_{t}=\frac{q_{e}^{2}\;\;k_{2}\;t}{q_{e}\;k_{2}\;t+1}$	$k_{2\;}$(g mg^−1^ min^−1^): rate constant of the second-order adsorption	[25]
Intra-particle diffusion	$q_{t}=k_{p}\;t^{1/2}+C$	$k_{p}\;\;$(mg·g^−1^ min^0.5^): intraparticle diffusion rate constant. *C* (mg·g^−1^): intercept of the line.	[26]

**Table 3:** Isotherms models used to fit the adsorption of MO on adsorbent Mn–mica.

Isotherm Model	Equation	Parameters	Refs.
Langmuir	$q_{e}=\frac{q_{\max\;K_{L}C_{e}\;}}{\left(1+K_{L}C_{e}\;\right)}$	$C_{e}$ (mg·L^−1^): equilibrium concentration of the MO in the solution $q_{e}\;$(mg·g^−1^): removed amount of MO at equilibrium. $q_{\max}$ (mg·g^−1^): maximum adsorption capacity $K_{L}$ (L·mg^−1^): Langmuir constant	[27]
Freundlich	$q_{e}=K_{F}$ $C_{e}{}^{1/n}$	$K_{F}$ (mg/g)/(mg/L)^1/n^: MO adsorption capacity. *n*: heterogeneity factor.	[28]
Dubinin–Radushkevich $R^{2}=1-\frac{\sum {\left(q_{e,\exp}-q_{e,\mathrm{cal}}\right)}^{2}}{\sum {\left(q_{e,\exp}-q_{e,mean}\right)}^{2}}$ $\chi^{2}=\sum \frac{{\left(q_{e,\exp}-q_{e,\mathrm{cal}}\right)}^{2}}{q_{e,\mathrm{cal}}}$	$q_{e}=q_{m}e^{-\beta \varepsilon^{2}}$	β(mol^2^/kJ^2^): D-R constant ε (kJ^2^/mol^2^):Polanyil potential, equal to $RT\ln\left(1+\frac{1}{C_{e}}\right)$. R: universal gas constant (8.31 J/mol·K).*T* (K): absolute temperature. $q_{m}$ (mg·g^−1^): theoretical adsorption capacity. $q_{e,\exp}:$ Experimental adsorption capacity (mg·g^−1^) $q_{e,\mathrm{cal}}$: Calculated adsorption capacity (mg·g^−1^)	[29]

**Table 4:** Parameters of kinetic models for the adsorption of MO on adsorbent Mn–mica.

Kinetic Model	*T =* 25 °C	*T =* 40 °C	*T =* 55 °C
**Pseudo-first-order**			
$q_{e(\exp)}\;$ (${\mathrm{mg}\cdot g}^{-1}$)	47.71	46.73	44.83
$q_{e(\mathrm{cal})}\;$ (${\mathrm{mg}\cdot g}^{-1}$)	44.096	45.659	44.171
$k_{1}\;$ (min^−1^)	0.149	0.039	0.029
$R^{2}$	0.9839	0.9797	0.9819
**Pseudo-second-order**			
$q_{e}\;$ (${\mathrm{mg}\cdot g}^{-1}$)	47.355	46.927	46.309
$k_{2}\;$ (g (mg·min)^−1^)	0.0033	0.0021	0.0013
$R^{2}$	0.9934	0.99	0.9883
**Intra-particle diffusion**			
$k_{p}$ (mg/g·min^0.5^)	1.102	1.252	1.371
*C* (mg/g)	28.109	24.258	19.878
$R^{2}$	0.743	0.7709	0.8036

**Table 5:** Parameters of isotherms models for the adsorption of MO on adsorbent Mn–mica.

Isotherm Model	*T = * 25 °C	*T = * 40 °C	*T = * 55 °C
**Langmuir**			
$q_{\max}\;$(${\mathrm{mg}\cdot g}^{-1}$)	107.298	97.36	92.76
$k_{L}\;$ (${L\cdot \mathrm{mg}}^{-1})$	0.044	0.018	0.012
$R^{2}$	0.9979	0.9975	0.999
$\chi^{2}$	1.36	0.762	0.301
**Freundlich**			
$k_{F}$ (mg/g)/(mg/L)^1/n^	9.726	3.894	2.426
1/n	0.532	0.628	0.679
$R^{2}$	0.9982	0.9971	0.9998
$\chi^{2}$	0.571	0.542	0.067
**D − R**			
$q_{m}\left({\mathrm{mg}\cdot g}^{-1}\right)$	140.255	107.442	91.642
$E\left({\mathrm{kJ}\cdot \mathrm{mol}}^{-1}\right)$	6.753	5.972	5.822
$R^{2}$	0.9985	0.9974	0.9989
$\chi^{2}$	0.716	0.731	0.309

**Table 6:** Steric and energetic parameters of the multilayer layer model for the adsorption of MO on adsorbent Mn-mica.

T (°C)	n	$D_{M}\;(\mathrm{mg}\cdot g^{-1})\;$	$1+N_{2}\;(\mathrm{mg}\cdot g^{-1})\;$	$-\Delta E_{1}\;(\mathrm{kJ}\cdot {\mathrm{mol}}^{-1})$	$-\Delta E_{2}\;(\mathrm{kJ}\cdot {\mathrm{mol}}^{-1})$	$Q_{sat}\;(\mathrm{mg}\cdot g^{-1})$
**25**	1.83	59.34	1.75	29.116	20.77	190.44
**40**	1.54	60.85	1.65	13.38	12.01	154.99
**55**	1.32	69.18	1.53	13.24	10.66	140.33
